# Sequence and functional analyses of *Haemophilus *spp. genomic islands

**DOI:** 10.1186/gb-2007-8-11-r237

**Published:** 2007-11-08

**Authors:** Mario Juhas, Peter M Power, Rosalind M Harding, David JP Ferguson, Ioanna D Dimopoulou, Abdel RE Elamin, Zaini Mohd-Zain, Derek W Hood, Richard Adegbola, Alice Erwin, Arnold Smith, Robert S Munson, Alistair Harrison, Lucielle Mansfield, Stephen Bentley, Derrick W Crook

**Affiliations:** 1 Clinical Microbiology and Infectious Diseases, NDCLS, University of Oxford, Headley Way, Oxford OX3 9DU, UK; 2 The Weatherall Institute of Molecular Medicine, University of Oxford, Headley Way, Oxford OX3 9DS, UK; 3 Departments of Zoology and Statistics, University of Oxford, South Parks Road, Oxford OX1 3TG, UK; 4 Department of Pathology, University of Oxford, Headley Way, Oxford OX3 9DU, UK; 5 Faculty of Medicine, University Technology MARA, Shah Alam, 40450, Malaysia; 6 Medical Research Council Laboratories, PO Box 273, Banjul, Gambia; 7 Seattle Biomedical Research Institute, University of Washington, Westlake Avenue North, Seattle, WA 98109, USA; 8 The Center for Microbial Pathogenesis in Nationwide Children's Research Institute and The Center for Microbial Interface Biology, The Ohio State University, Children's Drive, Columbus, OH 43205, USA; 9 OCDEM, Churchill Hospital, Old Road, Oxford OX3 7LJ, UK; 10 The Wellcome Trust Sanger Institute, Hinxton, Cambridge CB10 1SA, UK

## Abstract

**Background:**

A major part of horizontal gene transfer that contributes to the diversification and adaptation of bacteria is facilitated by genomic islands. The evolution of these islands is poorly understood. Some progress was made with the identification of a set of phylogenetically related genomic islands among the Proteobacteria, recognized from the investigation of the evolutionary origins of a *Haemophilus influenzae *antibiotic resistance island, namely ICE*Hin1056*. More clarity comes from this comparative analysis of seven complete sequences of the ICE*Hin1056 *genomic island subfamily.

**Results:**

These genomic islands have core and accessory genes in approximately equal proportion, with none demonstrating recent acquisition from other islands. The number of variable sites within core genes is similar to that found in the host bacteria. Furthermore, the GC content of the core genes is similar to that of the host bacteria (38% to 40%). Most of the core gene content is formed by the syntenic type IV secretion system dependent conjugative module and replicative module. GC content and lack of variable sites indicate that the antibiotic resistance genes were acquired relatively recently. An analysis of conjugation efficiency and antibiotic susceptibility demonstrates that phenotypic expression of genomic island-borne genes differs between different hosts.

**Conclusion:**

Genomic islands of the ICE*Hin1056 *subfamily have a longstanding relationship with *H. influenzae *and *H. parainfluenzae *and are co-evolving as semi-autonomous genomes within the 'supragenomes' of their host species. They have promoted bacterial diversity and adaptation through becoming efficient vectors of antibiotic resistance by the recent acquisition of antibiotic resistance transposons.

## Abstract 2

Comparative analysis of genomic islands of *Haemophilus *species shows that they are co-evolving as semi-autonomous genomes within the host genome.

## Background

Horizontal gene transfer contributes to the diversification and adaptation of micro-organisms. Apart from the core genes that are present in all strains of the same species and are the minimum necessary for survival under optimal growth conditions, bacterial genomes also harbor a variable number of accessory genes that are acquired by horizontal gene transfer, many of which are crucial for bacterial adaptation and survival. The sum of the core genes and accessory genes across the species represent what is regarded as the 'supragenome'. The accessory genes are frequently aggregated into heterogeneous sets of hitherto ill-defined structures called genomic islands, the origins of which are unknown. We recently reported on a set of genomic islands found among Proteobacteria that shared a common ancestor and were coherently structured [1]. This study suggested for the first time that a family of genomic islands had a deep evolutionary history. However, further evidence indicated they were also capable of propagation by self-directed transfer through conjugation and replication [2,3]. The relationships between species-specific subfamilies of this family of genomic islands found among Proteobacteria have not been determined.

In general, genomic islands, although poorly defined, have been regarded as segments of DNA acquired by horizontal gene transfer, with major features that include the following: GC content that is usually different from the rest of the genome; common insertion in tRNA genes; direct repeated DNA sequences at the ends; and the presence of genes such as integrases, transposases, or insertion sequences. Genomic islands often offer selective advantages; thus, according to their gene content, they can be described as pathogenicity, symbiosis, metabolic, fitness, or resistance islands [4]. Whether all genomic islands will be classified into related families remains to be seen.

The family of genomic islands we previously reported was identified through investigations into the origins of antibiotic resistance that emerged in *Haemophilus influenzae *in the early 1970s [5,6]. Determining the origins of antibiotic resistance focused on the sequence of an exemplar genomic island named ICE*Hin1056*. ICE*Hin1056 *was shown to belong to a family of genomic islands with deep evolutionary origins found among Proteobacteria, including *Yersinia enterocolitica*, *Salmonella enterica *serovar Typhi, *Pseudomonas fluorescens*, *Ralstonia metallidurans*, *Pseudomonas *sp. B13, and *Pseudomonas aeruginosa *[1,7-9]. These islands functioned as integrative and conjugative elements (ICEs). Conjugation, the process for self-directed transfer of elements between bacteria, is facilitated in this family of genomic islands by a process involving a novel type IV secretion system (T4SS) [2]. Furthermore, replication, transfer, and integration of these genomic islands into recipient strains of the host species has been demonstrated [2,3]. These limited data indicate a semi-autonomous existence for species-specific subfamilies of these genomic islands; however, further evidence is needed.

Despite an unknown potential for horizontal transfer between species, the islands we have studied [1,2] show striking evidence of a phylogeny shaped by descent within the deep evolutionary history of their host bacterial species. In particular, a single common ancestor can be recognized for genomic islands carried by *H. influenzae*, *Haemophilus ducreyi*, and *Haemophilus somnus*. The contrast between evidence for phylogenetic descent on the one hand [1] and for potential horizontal spread by conjugation on the other [2] raised questions about the factors both promoting and limiting conjugative spread of these genomic islands. A more thorough understanding of how these genomic islands are evolving and functionally behaving may explain the spread of antibiotic resistance in *H. influenzae*. Accordingly, a detailed comparative investigation of multiple examples of the ICE*Hin1056 *subfamily was undertaken.

To address the questions raised above, we report on sequence and functional analyses of seven genomic islands identified in *H. influenzae *and *Haemophilus parainfluenzae*. A set of shared core genes predicted to encode proteins involved in replication and T4SS-dependent transfer is described, and we show that these islands also carry a variety of accessory genes that contribute to genetic diversity. The extent and distribution of accessory gene content of this subfamily of genomic islands is similar to that reported for the overall supragenome of the bacterial host *H. influenzae*. The findings suggest substantial variation in sequence and gene content that is inconsistent with a historically recent acquisition and clonal expansion of these islands. However, the transposons containing antibiotic resistance genes exhibit features indicating that they have more recently been acquired. The conjugative and antibiotic susceptibility phenotypes are compared between islands and the observed variation is attributed to associated background diversity in the bacterial host genomes.

## Results and discussion

### General features of *H. influenzae *and *H. parainfluenzae *genomic islands

**Figure 1:**
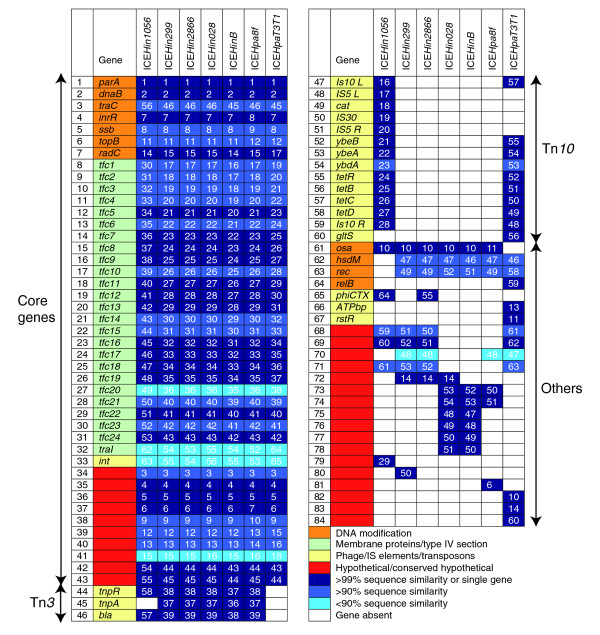
The gene content of *Haemophilus *spp. genomic islands. Forty-three genes, comprising mostly the putative replication and transfer modules, constitute an essential set of core genes for genomic islands of human oropharyngeal *Haemophilus *spp. Numbers (white) in the table represent the position of the gene in the respective genomic island. ICE, integrative and conjugative element; Tn, transposon.

At the time of analysis seven genomic islands were available for study. Three were individually sequenced, whereas the others were identified from completed bacterial genomic sequences (Table 1). The islands were found among *H. influenzae *and *H. parainfluenzae *as follows: ICE*Hin1056 *in *H. influenzae *1056, ICE*Hin299 *in *H. influenzae *299, ICE*Hin2866 *in *H. influenzae *2866, ICE*Hin028 *in *H. influenzae *028, ICE*HinB *in *H. influenzae *B, ICE*Hpa8f *in *H. parainfluenzae *8f, and ICE*HpaT3T1 *in *H. parainfluenzae *T3T1 (Table 1). General features of these *Haemophilus *spp. genomic islands and their gene content are shown in Table 2 and Figure 1, respectively. Among these, the *H. influenzae *island ICE*Hin1056 *and the *H. parainfluenzae *island ICE*HpaT3T1 *are the largest and have the most open reading frames. Only a single copy of the genomic island was identified per chromosome of the respective host strain (data not shown).

**Table 1:** Bacterial strains and genomic islands

Strain/genomic island	Characteristics/origin (year, where applicable)	Reference or source
Strain		
1056	*Hin *type b/UK (1990)	[36]
299	*Hin *type b/Greece (1991)	[37]
2866	*Hin*/USA (1994)	[38]
028	*Hin*/USA (1986)	[39]
HinB	*Hin *type b 10810/UK (2003)	Sanger Centre, UK
8f	*Hpa*/UK (1995)	Laboratory collection
T3T1	*Hpa*/Gambia (2001)	Sanger Centre, UK
Rd	*Hin *type d laboratory strain	[25]
Rd (ICE*Hin1056*)	Strain Rd harboring ICE*Hin1056*	The present study
Rd (ICE*Hin299*)	Strain Rd harboring ICE*Hin299*	The present study
Rd (ICE*Hin286*6)	Strain Rd harboring ICE*Hin2866*	The present study
Rd (ICE*Hpa8f*)	Strain Rd harboring ICE*Hpa8f*	The present study
Rd (ICE*HpaT3T1*)	Strain Rd harboring ICE*HpaT3T1*	The present study
Genomic island^a^		
ICE*Hin1056*	*Hin *1056 island	[1]
ICE*Hin299*	*Hin *299 island	[37]
ICE*Hin2866*	*Hin *2866 island	National Center for Biotechnology Information
ICE*Hin028*	*Hin *86-028NP island	[39]
ICE*HinB*	*Hin *B island	Sanger Centre, UK
ICE*Hpa8f*	*Hpa *8f island	Laboratory collection
ICE*HpaT3T1*	*Hpa *T3T1 island	Sanger Centre, UK

^a^Sequence available through accession number or online database: ICE*Hin1056*, AJ627386; ICE*Hin299*, AM884334; ICE*Hin2866*, AADP00000000, ICE*Hin028*, CP000057; ICE*HinB *[40]; ICE*Hpa8f*, AM884335; ICE*HpaT3T1 *[41]. *Hin*, *Haemophilus influenzae*; *Hpa *= *Haemophilus parainfluenzae*; ICE, integrative and conjugative element.

**Table 2:** General features of *Haemophilus *spp. genomic islands

GI^a^	Size (base pairs)	GC content (%)	Number of ORFs
ICE*Hin1056*	59,393	39.1	64
ICE*Hin299*	53,902	38.6	55
ICE*Hin2866*	53,114	38.7	55
ICE*Hin028*	55,345	38.6	56
ICE*HinB*	55,996	38.8	56
ICE*Hpa8f*	52,725	38.8	53
ICE*HpaT3T1*	60,056	37.8	65

^a^The GC contents of host bacterial genomes available are as follows: ICE*Hin2866*, 38.1%, ICE*Hin028*, 38.2%; ICE*HinB*, 38.1%; ICE*HpaT3T1*, 39.6%. Genomic islands are horizontally acquired segments of DNA, with major features that include: GC content different from the rest of the genome, insertion in tRNA genes, direct repeated DNA sequences at the ends, and the presence of mobility genes. Genomic islands that are capable of integration into the chromosome of the host, excision, and self-transfer to a new host and reintegration are usually called Integrative and Conjugative Elements (ICEs).

**Figure 2:**
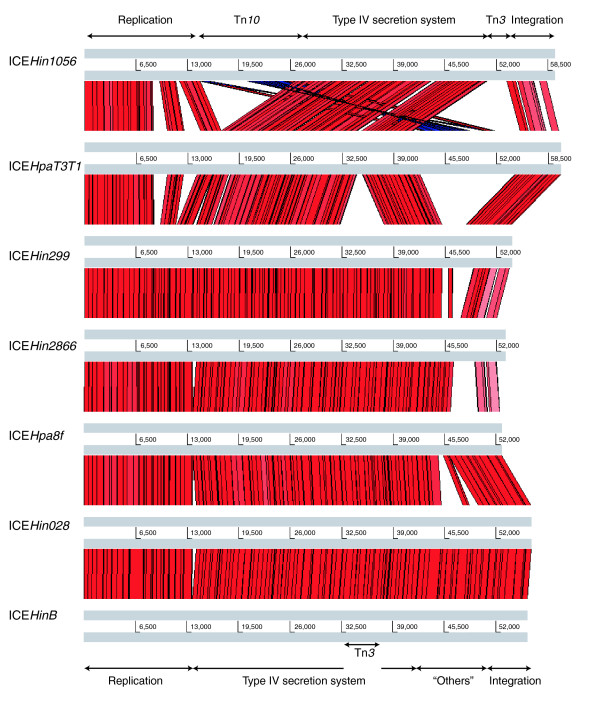
The Artemis comparison tool analysis of genomic island sequences. All genomic islands tested share extensive sequence homology, and the genes are arranged mostly in the same order. Red vertical lines correspond to regions that are highly homologous, mainly in the putative replication and type IV secretion system modules. Pink vertical lines (right end) correspond to regions with lower sequence homologies, in particular for the putative integration module. Blue vertical lines depict a region containing the transposon (Tn)*10*, whose orientation is reversed between ICE*Hin1056 *and ICE*HpaT3T1*. Arrows on top and bottom delimit the functional regions whose order is well conserved in all seven genomic islands. ICE, integrative and conjugative element.

All seven genomic islands exhibited extensive DNA sequence homologies and preserved gene order in most cases (Figure 1). The only major difference in gene order is represented by the region containing Tn*10*, in which the orientation is reversed between ICE*Hin1056 *and ICE*HpaT3T1 *(Figure 2). Whereas high sequence similarities (some of 100%) were observed, especially in the putative replication and T4SS modules (Figures 1 and 2), the degree of sequence conservation between pairs of genomic islands varied more widely in the putative integration module (right-hand side of Figure 2). For example, we observed 74% to 99% nucleotide sequence similarity for *int*, encoding the putative integrase.

As shown in Figure 1, a total of 84 open reading frames were identified in the seven genomic islands surveyed. The putative replication module and the putative transfer module (including T4SS) comprised the majority of the 43 genes conserved across all genomic islands. These 43 genes were defined to constitute the essential core set of genes of an ICE*Hin1056*-based subfamily of genomic islands. The remaining 41 genes, including the transposon-based genes, are likely to be accessory genes. A majority of the accessory genes, whether they were part of transposons (transposon [Tn]*3 *in ICE*Hin1056 *or Tn*10 *in ICE*HpaT3T1*) or not, were located 3' of *traC *and 5' to the putative *traI*. Many of these accessory genes have unknown functions. This localized site of accessory gene presence suggests that this is a region that is particularly permissive for their insertion. Curiously, this region in the larger family of related proteobacterial genomic islands is also a nidus for accessory gene insertion [1], suggesting that this area is a 'hot spot' for recombination for the entire family. No explanation for this phenomenon is apparent, although this region is just 5' to the putative *oriT *of ICE*Hin1056*.

The observed distribution between core genes and accessory genes in this subfamily of genomic islands indicates that 50% are accessory. This is remarkably similar to the distribution found in the complete *H. influenzae *genomes analyzed by Hogg and coworkers [10]. Furthermore, excluding the antibiotic resistance transposons, the GC content of the other accessory genes is similar to that of the *H. influenzae *genome and to that of these genomic islands' core genes. These features suggest that this subfamily of genomic islands conforms to the distributed genome hypothesis in the same way that the host genome does. This hypothesis proposes that the full complement of genes available to a pathogenic bacterial species exists in a supragenome pool, which is not contained by any particular strain. Although individual strains carry only a subset of genes, a diverse range of genes can be available to naturally transformable bacterial strains [10]. Evidence for the distributed genome hypothesis has thus far been demonstrated for several bacterial pathogens, including *H. influenzae*, *Streptococcus pneumoniae*, and *P. aeruginosa *[10-12].

### Diversity and co-evolution of the ICE*Hin1056 *sub-family of genomic islands with the host

The divergence in gene content between the seven genomic islands described here is clearly illustrated in Figure 1. No two islands were identical and they differed by many genes. Even the core genes of conserved modules exhibited many variable sites, with the nucleotide sequences between several core genes differing by up to 10% or more (Figure 1). A similar level of variation is seen in the housekeeping genes of the bacterial host *H. influenzae *[13]. These accumulations of mutations must have taken substantial time. Both the divergence in gene content between the genomic islands and the variation in nucleotide sequence of their core genes are contrary to these genomic islands having recently been acquired and disseminated by clonal expansion. Furthermore, the similarity of GC content of the genomic island core genes (38%) to the GC content of the host genomes (Table 2) suggests that this ratio for the island has ameliorated to that of the host bacteria, which is time dependent. All these features are consistent with this subfamily of genomic islands having a longstanding association with its *Haemophilus *host species.

None of these genomic islands contained nucleotide sequences indicating recent acquisition from other subfamilies present in *H. somnus *and *H. ducreyi*, nor from more distantly related members of this family of islands. This observation provides evidence that the ICE*Hin1056 *subfamily is evolving by descent within its host species without recombination with other species-specific subfamilies. However, it is curious that the two genomic islands present in *H. parainfluenzae *had no features to distinguish them specifically from the five genomic islands present in *H. influenzae*. From other recent work, this ICE*Hin1056 *subfamily of genomic islands appears to be shared only by these two human *Haemophilus *spp. [14]. The factors that limit this genomic island to two species to the exclusion of the many other closely related human *Haemophilus *spp. are unclear and await further investigation.

### Highly conserved putative replication module

The putative replication module of the genomic islands surveyed is composed of between 15 and 18 genes, mostly of hypothetical function, that share extensive sequence homology and are arranged in the same order. Fourteen of these genes, including homologs of *parA*, *dnaB*, *ssb*, *topB*, *inrR*, and *radC*, were present in all islands tested and would be predicted to be involved in DNA replication. The gene *parA *encodes the chromosome partitioning protein that divides and distributes plasmid copies upon cell division; *dnaB *encodes a helicase that unwinds DNA strands; *ssb *encodes a single-strand protein that binds the lagging DNA strand thus preventing re-annealing; *topB *encodes a topoisomerase that protects free DNA strands from random ligation; and *radC *encodes a DNA repair protein involved in the recombination repair associated with a DNA replication fork [15-17]. One of the other encoded conserved hypothetical proteins exhibited low similarity to the carboxyl-terminal region of *Shigella flexneri *OSA protein, which is known to suppress the oncogenicity activity in *Agrobacterium tumefaciens*, but its function in *H. influenzae *is unclear [18].

The most notable variation in the putative replication module was found in the *H. parainfluenzae *genomic island ICE*HpaT3T1*, where the *osa *homolog, which is present in all of the other islands, was missing and substituted by four ICE*HpaT3T1*-specific genes. Out of these four genes, two encode hypothetical proteins, whereas the others encode an ATP-binding protein that is highly conserved in variety of bacterial species and a possible filamentous phage CTX-RtsR-like repressor. The fact that ICE*HpaT3T1 *is missing the OSA homolog and that the four ICE*HpaT3T1*-specific genes are not present in other islands would suggest that only the remaining 14 genes are essential for the replication of this subfamily of genomic islands.

The putative origin of replication *oriV *has been identified in the replication module, based on the presence of iteron-like (multiple direct repeats of DNA bases) sequences and multiple indirect repeats in an AT-rich region [19]. These features are consistent with the *oriVs *of the larger family of genomic islands found in a variety of other Proteobacteria, including pKLC102 of *P. aeruginosa *C [20], SPI-7 of *S. enterica *serovar Typhi [7], and PAPI of *P. aeruginosa *PA14 [21].

### Highly conserved type IV secretion module

Apart from the putative replication module, the T4SS module was the most conserved part of the ICE*Hin1056 *subfamily of genomic islands, with DNA similarity ranging from 95% to 100% between islands. T4SSs are multi-subunit cell envelope spanning structures that comprise a secretion channel and often a pilus or other surface filament or protein [22]. Conjugation systems, assembled by a subfamily of the T4SSs, are usually used in the process of conjugative transfer of DNA from donor to recipient cells [23]. In our previous work we showed that this conserved cluster of genes encodes a novel T4SS that is responsible for formation of the conjugative pilus and the resulting conjugative transfer of the *H. influenzae *genomic island ICE*Hin1056 *[2].

**Figure 3:**
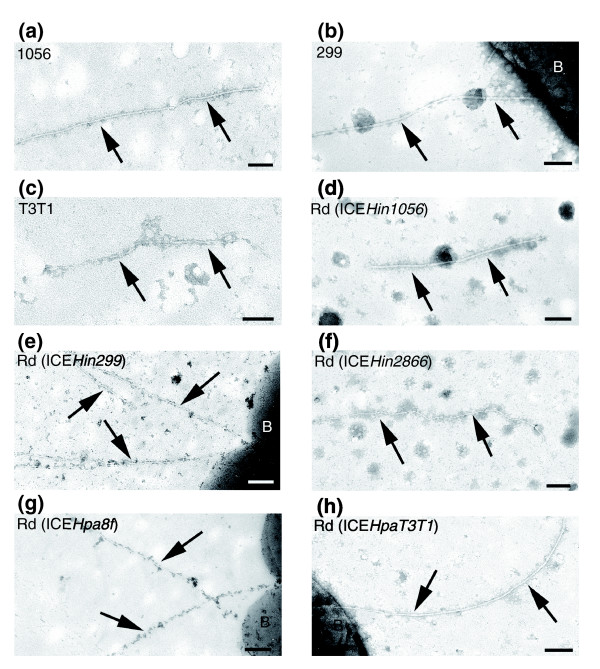
T4SS pili encoded by *Haemophilus *spp. genomic islands. Presented are transmission electron micrographs of negatively stained samples, showing presence of type IV secretion system (T4SS) dependent pili (arrows) in the *Haemophilus *spp. strains harboring genomic islands. Three parent *Haemophilus *spp. strains with the highest conjugation transfer frequencies harboring genomic islands **(a) **ICE*Hin1056*, **(b) **ICE*Hin299*, and **(c) **ICE*HpaT3T1 *were shown to produce the T4SS pilus. Moreover, *H. influenzae *strain Rd harboring genomic islands **(d) **ICE*Hin1056*, **(e) **ICE*Hin299*, **(f) **ICE*Hin2866*, **(g) **ICE*Hpa8f*, **(h) **and ICE*HpaT3T1 *produced T4SS pili, thus providing experimental evidence of the activity of T4SS encoded by these genomic islands. Bars represent 100 nm. B, bacterium; ICE, integrative and conjugative element.

Of the *Haemophilus *spp. strains harboring islands, only those that exhibited the highest conjugation transfer frequencies (ICE*Hin1056*, ICE*Hin299*, and ICE*HpaT3T1*) were shown to produce the T4SS pilus (Figure 3a-c and Table 3). It is reasonable to assume that this pilus is produced also by other genomic islands surveyed, but its presence is harder to detect because of their lower conjugation transfer frequencies (see below). This hypothesis was tested by an experiment in which different genomic islands were transferred into the same host background (*H. influenzae *strain Rd). The characterized *H. influenzae *fimbrial gene cluster (*hif*) is absent in strain Rd, which has not been demonstrated to produce either fimbriae or type IV pili [24,25]. However, electron microscopic examination of strain Rd harboring genomic islands ICE*Hin1056*, ICE*Hin299*, ICE*Hin2866*, ICE*Hpa8f*, and ICE*HpaT3T1 *revealed T4SS pili (Figure 3d-h), thus providing experimental evidence on the activity of T4SS encoded by these genomic islands.

**Table 3:** Conjugation frequencies of the *Haemophilus *spp. genomic islands from the given donor strain to strain Rd

Donor strain	Conjugation frequency^a^
1,056	6.5 × 10^-5 ^(± 2.8 × 10^-5^)
299	8 × 10^-5 ^(± 4.5 × 10^-5^)
2,866	3.3 × 10^-6 ^(± 1.3 × 10^-6^)
HinB	Not detectable
8f	≤ 10^-9^
T3T1	3.8 × 10^-6 ^(± 1.6 × 10^-6^)
Rd (ICE*Hin1056*)	3 × 10^-1 ^(± 0.6 × 10^-1^)
Rd (ICE*Hin299*)	1.7 × 10^-1 ^(± 0.3 × 10^-1^)
Rd (ICE*Hin2866*)	1.6 × 10^-1 ^(± 0.1 × 10^-1^)
Rd (ICE*Hpa8f*)	1.5 × 10^-1 ^(± 0.03 × 10^-1^)
Rd (ICE*HpaT3T1*)	3.9 × 10^-2 ^(± 0.7 × 10^-2^)

For conjugation frequencies the mean values from three independent experiments are given. Numbers in brackets represent standard errors. ^a^'Conjugation frequency' is the number of transconjugants divided by the number of recipients. ICE, integrative and conjugative element.

Further experimental proof of the activity of the T4SS encoded by the genomic islands tested was provided by the analysis of the conjugation transfer frequencies. As shown in Table 3, genomic islands were transferred from the donors to a *rec*^- ^streptomycin-resistant recipient *H. influenzae *strain (Rd) by conjugation, albeit with different efficiencies ranging from 10^-5 ^(ICE*Hin1056 *and ICE*Hin299*) to 10^-9 ^(ICE*Hpa8f*). Any contribution of natural transformation and homologous recombination to transfer of genomic island-borne genes is remote because the recipient was recombination deficient and previous work did not demonstrate such a process [26]. Transconjugants were selected on the plates containing either streptomycin plus ampicillin or streptomycin plus tetracycline, and recipients were selected on plates containing streptomycin. The conjugation frequency of the island ICE*HinB *was assumed to be lower than could be detected using our method. To test whether the differences in the conjugal transfer frequencies of the ICE*Hin1056 *subfamily of genomic islands were due either to the respective host strain or to the gene content of the island, five genomic islands (ICE*Hin1056*, ICE*Hin299*, ICE*Hin2866*, ICE*Hpa8f*, and ICE*HpaT3T1*) present in the streptomycin-resistant *H. influenzae *strain Rd background were each transferred to the same recipient: a nalidixic acid-resistant strain Rd. Although recombination proficient, previous unpublished work does not demonstrate any transfer of genomic island borne genes by transformation and homologous recombination in this setting. Transconjugants were selected on plates containing either nalidixic acid plus ampicillin or nalidixic acid plus tetracycline, and recipients were selected on plates containing nalidixic acid. As shown in Table 3, the conjugal transfer frequencies were almost constant when *H. influenzae *strain Rd was used as a donor, which is indicative of the different host strains introducing variations in conjugation efficiency in the initial experiment. This observation is consistent with the very high sequence homology of the T4SS module between the genomic islands tested.

**Figure 4:**
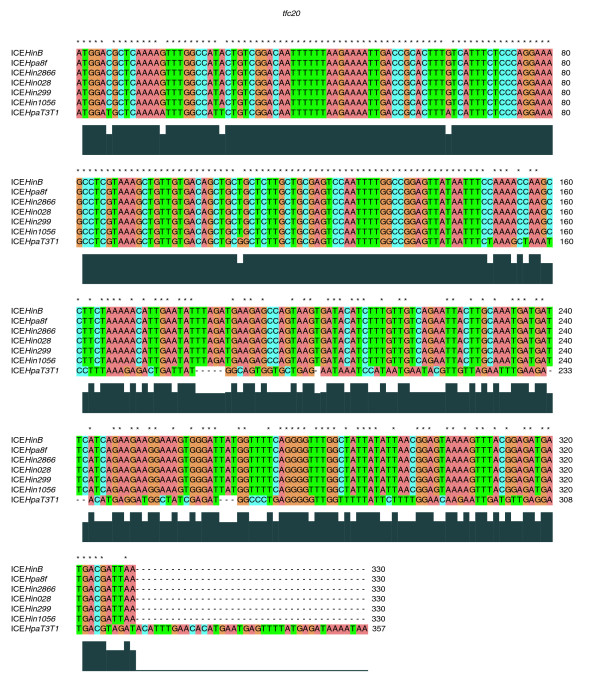
ClustalX alignment of nucleic acid sequences of gene *tfc20 *of the *Haemophilus *spp. genomic islands. *tfc20 *constitutes the only variation in the otherwise homogenous block of the conserved type IV secretion system module.

The only exception to similar conjugation frequencies in strain Rd was genomic island ICE*HpaT3T1*, which demonstrated a significantly reduced conjugation efficiency (Table 3). An explanation for the lower frequency of transfer of ICE*HpaT3T1 *from strain Rd donor, when compared with the others of the ICE*Hin1056 *subfamily, may be found in the sequence diversity of the gene*tfc20 *in ICE*HpaT3T1*. The *tfc20 *gene constitutes the only variation in the otherwise homogenous block of genes of the conserved T4SS module. The *tfc20 *gene encodes a hypothetical protein with unknown function that is conserved in six genomic islands surveyed. As shown in Figure 4, the second half of *tfc20 *in ICE*HpaT3T1 *differs substantially from *tfc20 *identified in the rest of the genomic islands surveyed. This may be a consequence of the insertion and subsequent excision of a transposon into and out of the ancestor of ICE*HpaT3T1*. Because Tn*3 *was found to be localized between genes *tfc20 *and *tfc21 *in five genomic islands, namely ICE*Hin299*, ICE*Hin2866*, ICE*Hin028*, ICE*HinB*, and ICE*Hpa8f*, the sequence between genes *tfc20 *and *tfc21 *may identify a 'hot spot' for transposon insertion.

### Variable antibiotic resistance genes

The genomic islands tested contain a range of antibiotic resistance genes, which is a crucial feature that contributes both to bacterial adaptation and to its clinical significance. Genes that confer resistance to three different antibiotics, namely ampicillin, tetracycline, and chloramphenicol, were identified. In each case, the antibiotic resistance gene determinants are carried on transposons, with ampicillin on Tn*3 *and tetracycline and chloramphenicol on Tn*10 *variants. The homologies of the transposon sequences were investigated in detail.

**Figure 5:**
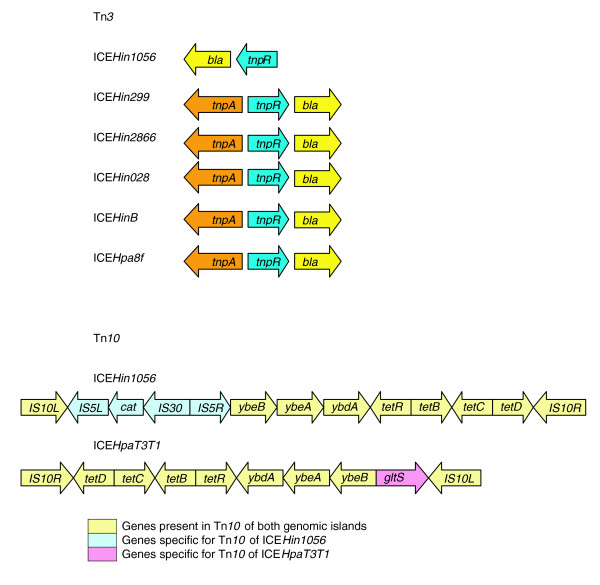
Antibiotic resistance gene content of *Haemophilus *spp. genomic islands. The genomic islands differ in their antibiotic resistance genes, which are located on two transposons, transposon Tn*3 *and Tn*10*. *bla*, β-lactamase; *cat*, chloramphenicol acetyltransferase; ICE, integrative and conjugative element; *tet*, tetracycline.

Tn*3 *is one of the most extensively studied and best understood of prokaryotic transposable elements. It consists of three genes: *tnpA*, which encodes a transposase that is essential for all transpositional recombination; *tnpR*, which encodes a resolvase that acts both as a site-specific recombinational enzyme and as a repressor of *tnpA *and *tnpR *expression; and *bla*, which encodes a β-lactamase precursor [27]. Tn*3 *was identified in six of the genomic islands tested, ICE*HpaT3T1 *being the exception. The complete copy of Tn*3 *revealed a gene organization identical to the *Escherichia coli *derived Tn*3 *transposon [28] and was present in ICE*Hin299*, ICE*Hin2866*, ICE*Hin028*, ICE*HinB*, and ICE*Hpa8f *(Figure 5). Correspondingly, the host strains harboring genomic islands ICE*Hin299*, ICE*Hin2866*, ICE*HinB*, and ICE*Hpa8f *were resistant to ampicillin (Table 4). Moreover, when these islands were transferred into the ampicillin-sensitive *H. influenzae *strain Rd, they also expressed ampicillin resistance; however, most exhibited much greater resistance than the parent strains (Table 4). A truncated Tn*3 *present in ICE*Hin1056 *did not contain the transposase *tnpA *(Figure 5). As shown in Table 4, truncation of Tn*3 *in ICE*Hin1056 *did not abolish the ampicillin resistance activity of the host strain. The reason for the truncation of Tn*3 *in ICE*Hin1056 *is unknown, although such modified transposons have frequently been identified in other bacteria. Tn*3 *is inserted in the same specific site, between genes *tfc20 *and *tfc21*, in the five genomic islands that harbor the complete copy. Moreover, the nucleotide sequences of *tnpA*, *tnpR*, and *bla *genes of these genomic islands were identical. ICE*Hin1056 *shares an identical *bla *gene, but it has a single variable site in its *tnpR *nucleotide sequence. The Tn*3 *found in *E. coli *[28] contained approximately 300 variable sites (1% to 6% difference depending on the gene) compared with those complete Tn*3*s found in the five *Haemophilus *spp. genomic islands. In addition the GC content of the Tn*3 *genes (50%) differed substantially from the core genes of this subfamily of the genomic islands (38%).

**Table 4:** Resistance to ampicillin, chloramphenicol, and tetracycline of the *Haemophilus *spp. genomic islands in the strains tested

Strain	Antibiotic resistance (μg/ml)		
	
	Ampicillin	Chloramphenicol	Tetracycline
1056	24	48	32
299	>256	0.5	0.5
2866	8	0.75	0.5
HinB	24	0.75	0.38
8f	24	0.75	0.5
T3T1	0.38	0.5	>256
Rd (ICE*Hin1056*)	48	24	48
Rd (ICE*Hin299*)	>256	0.5	0.75
Rd (ICE*Hin2866*)	>256	0.5	0.75
Rd (ICE*Hpa8f*)	>256	0.5	0.75
Rd (ICE*HpaT3T1*)	0.25	0.5	>256

ICE, integrative and conjugative element.

Tn*10 *is a composite bacterial transposon, consisting of indirectly repeating insertion sequences (ISs) IS*10*-right and IS*10*-left flanking genes, which are involved in tetracycline resistance [29]. Tn*10 *was identified in only two genomic islands, ICE*Hin1056 *and ICE*HpaT3T1*. In ICE*Hin1056 *Tn*10 *was a variant containing additional sequences incorporating IS*5*-right and IS*5*-left, which flank a chloramphenicol acetyltransferase gene (*cat*) and IS*30*. Correspondingly, ICE*Hin1056 *confers resistance to both chloramphenicol and tetracycline (Table 4). Moreover, ICE*Hin1056 *Tn*10 *was missing the *jemA/gltS *homolog, encoding sodium-dependent glutamate permease [30]. Absence of the *jemA/gltS *homolog suggests that ICE*Hin1056 *depends on *tetB *as the only transport system for tetracycline. This observation may also explain the quantitative difference in the level of tetracycline resistance conferred by ICE*Hin1056 *and *jemA/gltS *homolog-containing ICE*HpaT3T1 *(Table 4). Not only did the structure of the two Tn*10 *structures differ, but so did their sites of insertion and orientation. Furthermore, the GC content of the genes within Tn*10 *were as follows: 45% for *IS10L*, 52% for *IS5L*, 42% for *cat*, 55% for *IS30*, 51% for *IS5R*, 41% for *ybdA*, 40% for *tetR*, 43% for *tetB*, and 45% *IS10R*. These differed substantially from the GC content of the genomic island core genes (38%).

These data suggest that these genomic islands acquired Tn*3 *and Tn*10 *a number of times, both independently and recently. It is not possible to estimate the dates of these events. At the latest it was approximately 1972 when antibiotic resistance was first detected in *H. influenzae*. Additional lines of evidence indicating recent acquisition are as follows: first, the lack of sequence diversity between complete Tn*3 *sequences found in five genomic islands tested; and second, the different GC content compared with that for the genomic island core genes and for the host chromosome, indicating a lack of time dependent amelioration. Although only two examples of Tn*10 *were available for study, their distinct and higher GC content is also consistent with relatively recent acquisition. The lack of sequence diversity among the complete Tn*3 *sequences and their identical point of insertion are curious, because they are from bacterial isolates obtained from points separated markedly in time and geography (Table 1). This may suggest that the same variant of Tn*3 *is being repeatedly acquired from an independent source and inserting at a 'hot spot' in this subfamily of genomic islands. Alternatively, it may be that Tn*3 *was acquired as a rare event and has disseminated rapidly worldwide by conjugative transfer of the host genomic island. We are currently actively investigating this possibility. Whichever interpretation is confirmed, it can be inferred that the ICE*Hin1056 *subfamily of genomic islands are small self-perpetuating genomes that are dependent both on a set of highly conserved core genes and on their host bacterial species. However, this subfamily is also the nidus for the accumulation of antibiotic resistance genes, making these islands vectors for the dissemination of antibiotic resistance and thus promoting survival of the bacterial host species in the presence of antibiotic selective pressures.

## Conclusion

Our analysis clearly shows that the ICE*Hin1056 *subfamily of genomic islands is diverse and has not recently emerged in *Haemophilus *spp., for example in association with the emergence of antibiotic resistance. Gene content and distribution between core and accessory genes of the seven genomic islands surveyed is remarkably similar to the host *H. influenzae *'supragenome' and conforms to the distributed genome hypothesis. From analysis of individual members' core genes, it can be concluded that this subfamily of semi-autonomous structures is co-evolving with the supragenomes of two highly related host species: *H. influenzae *and *H. parainfluenzae*. Although mobile between host bacterial strains, the observation of host specificity for the subfamily of genomic islands investigated is likely to be the case also for other subfamilies found in Proteobacteria.

The core genes are organized in modules with conserved gene order that encode functions that are crucial to the propagation and survival of this subfamily of genomic islands. Almost identical gene organization is consistently preserved for these modules in the larger family of genomic islands distributed among many different Proteobacteria. This conservation suggests that these genes are functionally constrained and, acting in concert, they play a major role in the successful propagation and survival of these genomic islands within their bacterial hosts' supragenomes. Their importance is overtly manifested by the rapid dissemination of antibiotic resistance worldwide among *H. influenzae *and *H. parainfluenzae*. The recent acquisition of antibiotic resistance genes and the rapid global spread of antibiotic resistance over the past 30 to 40 years is a good illustration of how genomic islands contribute to bacterial diversification and adaptation. Furthermore, results presented here show that variations in the conjugative and antibiotic resistance phenotypes can be influenced by genomic differences in the host bacteria. These results suggest that host bacterial genes modify phenotypic expression of genomic island-borne genes. This work is likely to represent a paradigm for a much broader spectrum of genomic islands than the subfamily described here.

## Materials and methods

### Bacterial strains, genomic islands, and growth conditions

All bacterial strains and genomic islands used in this study are listed in Table 1. Unless stated otherwise, *H. influenzae *and *H. parainfluenzae *strains were grown on heart infusion broth (HIB) medium (Columbia agar containing nicotinamide adenine dinucleotide [15 μg/ml] and hemin [15 μg/ml]). When required, the media was supplemented with kanamycin (10 μg/ml), tetracycline (2 μg/ml), ampicillin (4 μg/ml), streptomycin (20 μg/ml), or nalidixic acid (10 μg/ml). All *Haemophilus *spp. plate cultures were grown for 24 to 48 hours at 37°C in an atmosphere containing 5% carbon dioxide. For liquid culture, *Haemophilus *spp. were grown in brain heart infusion broth (BHI) supplemented with nicotinamide adenine dinucleotide (10 μg/ml), hemin (15 μg/ml) and, where necessary, with antibiotics in the concentrations described above and incubated at 200 rpm on a rotatory shaker at 37°C.

### Antimicrobial susceptibility

Minimum inhibitory concentrations of ampicillin, chloramphenicol, and tetracycline were determined using E-test. The E-tests were performed using E-strips (AB Biodisk, Solna, Sweden), following the manufacturer's instructions and interpreted according to the manufacturer's guidelines.

### Conjugal transfer of the *Haemophilus *spp. genomic islands

The transfer efficiency of each genomic island was determined following a modification of the method proposed by Stuy [31]. Donor or recipient cells were grown for 48 hours on HIB agar and approximately 10^8 ^cells were scraped off the plate and re-suspended in 1 ml BHI broth. Ten microliters of the suspension of donor cells and 100 μl of the suspension of recipient cells were gently mixed, achieving a ratio of donor to recipient cells of approximately 1:10. This mixture was subsequently spread in the centre of antibiotic-free HIB agar plates, allowed to dry, and then incubated for 6 hours. The cells were then harvested by flooding the plate with 1 ml BHI broth and re-suspending using a spreading paddle. Serial dilutions were plated to determine the viable counts and the number of transconjugants, donors, and recipients using agar containing the appropriate selective antibiotic. In the first step (transfer of genomic islands from original host strains to a streptomycin-resistant recipient *rec*^-^*H. influenzae *strain Rd, using a method previously described [26]), transconjugants were selected on the plates containing either streptomycin plus ampicillin or streptomycin plus tetracycline, and recipients were selected on plates containing streptomycin. In the second step (transfer of genomic islands from streptomycin-resistant donor *H. influenzae *strain Rd to a nalidixic acid-resistant recipient *H. influenzae *strain Rd), transconjugants were selected on plates containing either nalidixic acid plus ampicillin or nalidixic acid plus tetracycline, and recipients were selected on plates containing nalidixic acid. Experiments were carried out in triplicate, and the mean values for conjugation frequency and its standard error for each strain were calculated. Conjugation frequencies were calculated as number of transconjugants divided by the number of recipients.

### Electron microscopy

To visualize the T4SS-dependent pilus, *Haemophilus *spp. were grown overnight and then allowed to conjugate on antibiotic-free HIB agar plates for 6 hours. The conjugating cells were harvested by flooding the plate with 1 ml distilled water and subsequently re-suspended using a spreading paddle. A copper grid coated with formvar and carbon was floated in this suspension for 2 minutes. Excess fluid from the copper grid was removed, prior to negative staining with 1% methyl tungstate. The cells were examined for the presence of a pilus by transmission electron microscopy.

### DNA sequence analysis

*H. influenzae *strain 299 and *H. parainfluenzae *strain 8f were sequenced by the method described earlier [1]. DNA sequence similarity searches for genomic islands related to ICE*Hin1056 *using the BLASTN and BLASTX algorithms [32], and position-specific iterated BLAST (PSI-BLAST) [33] were performed by interrogating databases available through the National Center for Biotechnology Information website. *Haemophilus *spp. genomic islands were annotated and visualized with Artemis [34], a sequence viewer and annotation tool that allows visualization of sequence features as well as the results of analyses within the context of the sequence, and its six-frame translation. Complete host genome sequences and sequences of genomic islands were compared using the TBLASTX algorithm of the Artemis comparison tool [34] to identify regions of homology. Comparison of both the nucleotide and translated protein sequences of the seven genomic islands included several different pair-wise combinations of sequences to ensure fair and unbiased comparison. Pair-wise alignment for the percentage of sequence homology between genomic islands tested was carried out using BLAST2 [35].

## Abbreviations

BHI, brain heart infusion broth; HIB, heart infusion broth; ICE, integrative and conjugative element; IS, insertion sequence; T4SS, type IV secretion system; Tn, transposon.

## Authors' contributions

MJ, DWH, and DWC designed the study. IDD, AREE, M-ZZ, RA, AE, AS, SB, LM, RSM, and AH sequenced genomic islands. MJ, PMP, and RMH performed sequence analyses. MJ, IDD, and DJPF performed functional analyses. MJ, PMP, RMH, and DWC evaluated the results. DWC supported the work. MJ and DWC wrote the paper.
